# Occupational Therapy Interventions for Dementia Caregivers: Scoping Review

**DOI:** 10.3390/healthcare10091764

**Published:** 2022-09-14

**Authors:** Alberto Martínez-Campos, Laura-María Compañ-Gabucio, Laura Torres-Collado, Manuela Garcia-de la Hera

**Affiliations:** 1 Unidad de Epidemiología de la Nutrición (EPINUT), Departamento de Salud Pública, Historia de la Ciencia y Ginecología, Universidad Miguel Hernández (UMH), 03550 Alicante, Spain; 2 Instituto de Investigación Sanitaria y Biomédica de Alicante, ISABIAL, 03010 Alicante, Spain; 3 CIBER Epidemiología y Salud Pública (CIBERESP), Instituto de Salud Carlos III, 28034 Madrid, Spain

**Keywords:** occupational therapy, caregivers, older adults, Alzheimer’s disease, home

## Abstract

Background and purpose: caregivers of people with dementia (PwD) often experience a significant caregiver burden. Occupational Therapy (OT) is a helpful discipline for improving quality of life and other health factors for these caregivers. We conducted a Scoping Review to describe OT interventions for caregivers of PwD. Methods: two authors searched PubMed, Scopus, EMBASE and Web of Science databases and OT journals indexed in the Journal Citation Reports. Terms included in the search strategy were: dementia, Alzheimer, Parkinson, caregivers and OT. We included articles with experimental design in which an OT intervention in caregivers of PwD was carried out, written in Spanish or English and with the full text available. Results: a total of 2121 articles were obtained, 31 of which were included; 22 of them described home-based OT interventions: Tailored Activity Program (TAP) (*n* = 5), Environmental Skill-Building Program (ESP) (*n* = 4) and Advancing Caregiver Training (ACT) (*n* = 3) and other household interventions (*n* = 10); the remaining studies described OT interventions in other settings (*n* = 9). Conclusions: OT interventions for caregivers of PwD were mainly carried out at home. The most commonly used interventions were TAP focused on caregivers of people with Alzheimer’s disease, aimed at lessening the burden, depression and stress experienced by caregivers.

## 1. Introduction

Dementia is a collective term for progressive brain syndromes that affect memory, thinking, behavior and emotions [1]. According to the World Health Organization (WHO), dementia affects more than fifty million people worldwide and it is estimated that between 5% and 8% of the general population aged 60 or more suffer from dementia during their lives [2]. Alzheimer’s is the most frequent type of dementia and represents 50–60% of all cases [1]. Dementia progressively limits the patient’s daily life activities [1], making them dependent on a caregiver. Thus, dementia is the main cause of disability and dependence among the elderly [1,2].

The greatest cost of this disease is on the human level; the families and caregivers of dementia patients suffer the main psychological, physical, social and financial consequences of this disease [3]. An expert report by the Spanish Foundation for Neurological Diseases shows that these consequences make necessary significant adjustments in every aspect of family life [3]. As the disease evolves, the patient’s functionality deteriorates, increasing the pressure on the caregiver leading to what is known as burnout syndrome [3]. This syndrome results in a high level of stress and anxiety which is present in more than 75% of caregivers [4]. In addition, caregivers tend to have high levels of depression, somatic symptoms, sense of social isolation, as well as less time to carry out daily life activities [5].

There is a great number of interventions carried out in caregivers of dementia patients by different disciplines to reduce burnout syndrome [6]. These interventions aim to provide caregivers with wellbeing and coping skills [6], care-centered programs (such as family respite) [7], mixed or multimodal programs (combining several interventions) [7] or physical activity, such as yoga or hydrotherapy [8]. Madruga et al. have shown that there is an inverse relationship between physical exercise and the level of burnout in caregivers [9]. Moreover, Kuo et al. carried out an environmental intervention which introduced changes in the home such as the elimination of barriers and reduction in stimuli in the home environment [10]. This encourages patient-caregiver coexistence, reduces depression and improves quality of life in the intervention group compared to the control group [10]. Similarly, Signe et al showed that psychosocial intervention decreases the caregivers’ frustration with patients’ problems and increases their level of satisfaction [11].

The effectiveness of occupational therapy stands out [12] among the interventions for dementia caregivers. Occupational therapy intervention involves educating the caregiver in how to compensate for the patients’ cognitive decline, how to improve the burden of care and how to strengthen coping behaviors to manage dependence [13]. In this sense, a previous study showed that training in problem solving skills and educational intervention reduces stress in caregivers [14]. In addition, Dooley and Hinojosa carried out an intervention in which occupational therapists gave caregivers recommendations for community-based assistance in the care of patients, showing favorable results on caregiver burden and quality of life [15].

A previous study has observed that occupational therapy interventions based on compensatory and environmental strategies in the home of patients and their caregivers which are carried out in addition to usual clinical care, are more successful than usual clinical care [16]. Considering that more than 16.2 million caregivers are affected by the indirect effects of dementia [17] and that occupational therapy intervention in this population could be beneficial, we carried out this scoping review with the aim to describe occupational therapy interventions for caregivers of people with dementia which have been studied in existing scientific literature. Specifically, we want to answer the question: which OT interventions performed in caregivers of people with dementia have been studied in existing scientific literature?

## 2. Materials and Methods

We performed a peer scoping review following the Cochrane Handbook guidelines (Version 6.2, 2021) [18] and the recommendations from the PRISMA report for scoping reviews (PRISMA-SCR) [19]. We have carried out this scoping review following four main steps: 1. Literature search, 2. Screening of articles and selection of studies, 3. Data extraction and 4. Synthesis of results.

### 2.1. Search Strategy

On 9 November 2021, we consulted several of the most recommended databases in the reviews according to the article by Bramer et al. [20]: PubMed, Scopus, EMBASE and Web of Science (WOS). In addition, a manual search was carried out in OT journals indexed in the Journal Citation Reports (JCR): American Journal of Occupational Therapy (AJOT); Australian Occupational Therapy Journal (AOTJ); British Journal of Occupational Therapy (BJOT); Canadian Journal of Occupational therapy (CJOT); Hong Kong Journal of Occupational therapy (HKJOT); Journal of Occupational Rehabilitation (JOR); Occupation, Participation, and Health (OTJR); Occupational Therapy International (OTI); Physical & Occupational Therapy In Pediatrics (POTP); Scandinavian Journal of Occupational Therapy (SJOT). We used the same search terms combined with the Boolean operators OR and AND in all the information sources consulted. The search terms used were “Alzheimer”, “dementia”, “Parkinson”, “caregiver” and “occupational therapy”. All of the search strategies can be found in Table 1. We did not use time limits in the literature search in any of the journals and databases consulted.

### 2.2. Review Criteria

We included articles that met the following criteria:

- Experimental studies (randomized or non-randomized intervention studies, exploratory studies, pilot studies and quasi-experimental studies). Rationale: we have included these study designs because they provide us with the greatest number of intervention characteristics, and therefore contribute to answering our research question. We have only included experimental studies at Joanna Briggs Institute (JBI) levels of evidence 1 and 2 [21] seeking to include only those study designs with the highest level of evidence;
- Experimental studies in which an occupational therapy intervention for caregivers (over 18 years old) of people with dementia (Alzheimer’s disease, Lewy body dementia, frontotemporal dementia, vascular dementia, Huntington’s disease, Pick’s disease dementia, Parkinson’s disease) was carried out. Rationale: caregivers of people with dementia should be included in the study population, as the aim of our study is to describe occupational therapy interventions directed at them;
- Experimental studies written in English or Spanish. Rationale: English is the most widely used language in research and therefore the language through which we can obtain most information in relation to our study objective. Although it is unusual to find articles published only in Spanish, we have added this language since it is our native language and if necessary, we will be able to complement the articles in English.

We excluded those articles where we were unable to obtain the full text, because the full text is necessary to carry out the full screening of the article or data extraction.

### 2.3. Study Selection

The titles of the search results obtained from the different sources of information consulted were downloaded onto a Microsoft Excel spreadsheet. From this Excel record, two authors independently carried out a complete screening of all the articles found. To facilitate the screening and reduce the subjectivity of the decisions to exclude or include an article, we created a table in Excel with the exclusion criteria for each screening phase. Accordingly, both authors marked the criteria that the articles did not meet in the table, thus specifying whether or not they would be included in the next screening phase. Screening consisted of elimination of duplicate articles (L.T.-C.) and subsequent screening by title, abstract and full text (L.-M.C.-G.); and (A.M.-C.). After each screening phase, the authors (L.T.-C., L.-M.C.-G.); and (A.M.-C.) met to compare their decisions and when two disagreed (L.-M.C.-G.); and (A.M.-C.), the third author (L.T.-C.) took the final decision.

### 2.4. Data Extraction and Synthesis

All three authors participated in data extraction and synthesis. Before data extraction, they prepared the three tables to be completed, following the recommendations in chapter 5 of the Cochrane Handbook [22]. The first table contains the main characteristics of the studies included in this review and comprises the following items: author, year, country, study design, sample, participants, intervention/comparator, assessment and study outcomes. The second table contains the main characteristics of the interventions carried out in the included studies and comprises the following items: author, year, participants, diagnosis, intervention activities, duration of intervention, sessions, professionals involved and main results. The third table contains the main limitations, funding sources and conflicts of interest declared in the included studies.

We performed a descriptive synthesis of the results. Wherever possible, we used tables and figures to present the study selection process and the characteristics of the studies included in this review. As a multidisciplinary research team, we discussed the classification categories of the different interventions carried out from occupational therapy among caregivers of people with dementia in the included studies, as well as how to structure the results section in this review. This was performed in order to reduce subjectivity in the process of synthesizing the information extracted from the studies included in this review.

### 2.5. Quality Assessment

The quality of the included studies was not critically assessed, as this is not a mandatory requirement of scoping reviews [23]. However, we made a qualitative synthesis in the results section of the limitations that the authors stated in their articles to help readers interpret the results of this review more objectively. In this sense, we reviewed the limitations section of each of the included articles and elaborated a table with this information that is included in our results section.

## 3. Results

**Figure 1:**
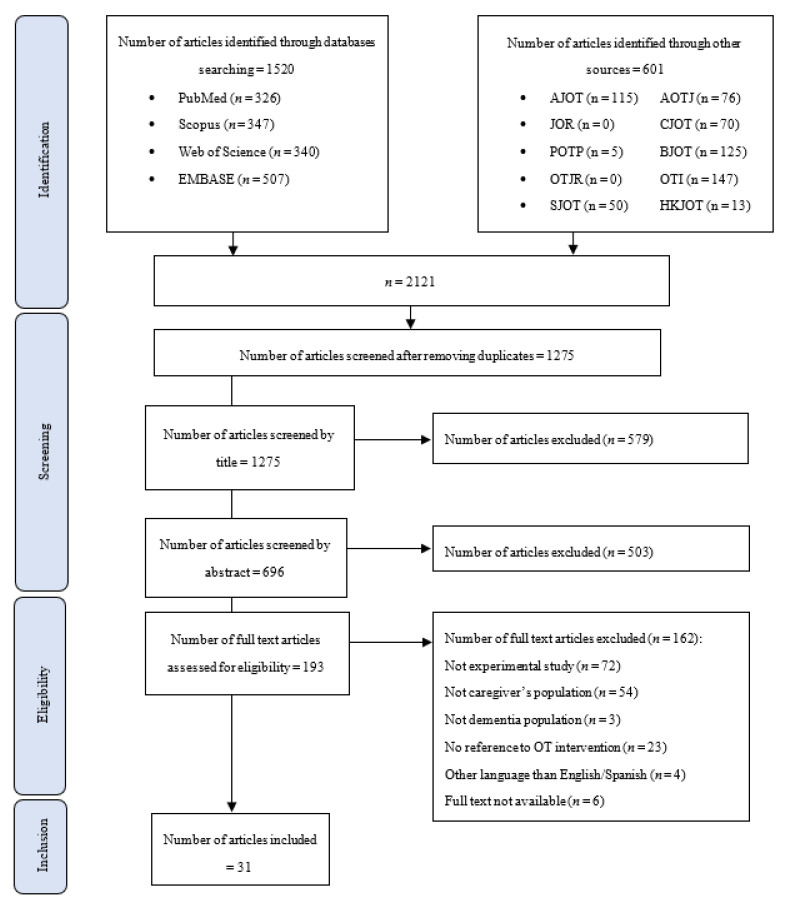
Flowchart of the study selection. AJOT, American Journal of Occupational Therapy; AOTJ, Australian Occupational Therapy Journal; BJOT, British Journal of Occupational Therapy; CJOT, Canadian Journal of Occupational Therapy; JOR, Journal of Occupation Rehabilitation; HKJOT, Hong Kong Journal of Occupational Therapy; OTI, Occupational Therapy International; OTJR, Occupation, Participation, and Health; POPT, Physical and Occupation Therapy in Pediatrics; SJOT, Scandinavian Journal of Occupational Therapy.

We identified a total of 2121 articles. After removing duplicates, 1275 articles remained for screening. We performed a three-stage screening, by title, by abstract and by full text, discarding 579 articles, 503 articles and 162 articles, respectively. Finally, 31 articles were included in this scoping review (Figure 1).

### 3.1. Main Characteristics of the Included Studies

More than half of the studies (48.4%) were conducted in the United States (*n* = 15) [14,24,25,26,27,28,29,30,31,32,33,34,35,36,37]. The remaining studies were conducted in the Netherlands (*n* = 4) [38,39,40,41], China (*n* = 3) [42,43,44], Australia (*n* = 3) [45,46,47], Italy (*n* = 2) [48,49], Brazil (*n* = 2) [50,51], Germany (*n* = 1) [52], and United Kingdom (*n* = 1) [53]. The majority of the studies (61.3%), were randomized clinical trials (*n* = 19) [27,28,29,30,31,33,34,36,37,38,39,40,41,42,44,47,49,52,53]. We found a smaller number of pilot studies (*n* = 6) [24,32,46,48,50,51], quasi-experimental studies (*n* = 3) [25,26,45], and non-randomized clinical trials (*n* = 3) [14,35,43] (Table S1).

### 3.2. Study Population in the Included Studies

In 64.5% of the articles, the study population consisted of a patient-caregiver dyad (*n* = 20) [24,32,33,34,36,38,39,40,42,43,44,45,46,47,48,49,50,51,52,53] and 35.5% comprised only caregivers of people with dementia (*n* = 11) [14,25,26,27,28,29,30,31,35,37,41]. In 67.7% of the articles (*n* = 21), the patients suffered from dementia, while in the remaining articles the type of dementia was specified as Alzheimer’s disease (*n* = 8) [24,27,28,30,35,37,49,52], Frontotemporal dementia (*n* = 1) [46] or Parkinson’s disease (*n* = 2) [40,41] (Table S1).

### 3.3. Main Intervention Characteristics of the Included Studies

In 74.2% of the studies, different interventions were carried out in the control and intervention groups (*n* = 23) [27,28,29,30,31,32,33,34,36,37,38,39,40,41,42,44,46,47,48,50,51,52,53]. Among these studies, the following comparisons stand out: Home Environmental Skill-building Program (ESP) vs. general counselling [28,29,30,31], Tailored Activity Program (TAP) vs. usual care [32,36,46,50,51], Advancing Caregiver Training (ACT) program vs. usual treatment [34,37], home-based occupational therapy intervention vs. usual care [38,39,40,41], home environmental interventions vs. usual treatment [27,33], active psychoeducation intervention vs. passive psychoeducation [42], dementia care education and activity programming vs. caregiver education [44,45], structured intervention vs. general counselling [48], community occupational therapy consultation vs. community occupational therapy in dementia program (COTiD) [52], telehealth vs. home visits [47], and COTiD vs. usual care [53] (Table S1).

In 25.8% of the articles, only one type of intervention was carried out (*n* = 8) [14,24,25,26,35,43,45,49]. These articles included interventions such as home environment intervention program [14,24,25], training for family caregivers [35], home incident prevention program [43], memory-making program [45], psychoeducational intervention for caregivers [49] and structured stress management course [26] (Table S1).

In all of the included articles, the occupational therapist was included in the multi-, inter- or trans-disciplinary team. In most of the articles, the occupational therapist lead the intervention (*n* = 22) [14,25,26,27,28,29,30,31,32,36,37,38,39,40,41,44,45,46,50,51,52,53], and in the rest of the articles (*n* = 9) [24,33,34,35,42,43,47,48,49] they developed the intervention together with other professionals such as nurses, psychologists, psychiatrists, clinical gerontologists or educators (Table S2). However, the occupational therapy intervention was not described in detail.

### 3.4. Occupational Therapy Interventions for Caregivers

Occupational therapy interventions for caregivers of people with dementia can be divided into two groups: home-based interventions (*n* = 22) [14,24,25,27,28,29,30,31,32,33,34,35,36,37,38,39,40,41,43,46,50,51] and other interventions (*n* = 9) [26,42,44,45,47,48,49,52,53] (Tables S1 and S2).

### 3.5. Home-Based Intervention: Tailored Activity Program (TAP)

TAP was the most commonly used occupational therapy intervention (*n* = 5) [32,36,46,50,51] in the included studies. TAP consisted of 3 phases. In the first phase, the occupational therapist conducts standardized assessments of the caregiver, patient and environment and, together with the caregiver, identifies three activities of interest to the people with dementia. In the second phase, the occupational therapist teaches the caregiver how to implement the activities through different strategies, such as role-playing. In the third phase, the occupational therapist helps the caregiver to generalize activity strategies to other caregiving challenges and helps to simplify communications and modify the environment. TAP lasted approximately three to four months and was divided into eight sessions lasting a maximum of one and a half hours each [32,36,46,50,51]. In all five studies, TAP was performed exclusively by the occupational therapist. Only one of them stated that they were treating a specific dementia, frontotemporal dementia [46].

### 3.6. Home-Based Intervention: The Environmental Skill-Building Program (ESP)

ESP was the second most used occupational therapy intervention (*n* = 4) [28,29,30,31] in the included studies. ESP was designed to increase caregiver mastery by introducing strategies to modify the physical, social and task dimensions of the home environment [30]. In the first ESP session, the occupational therapist conducted a needs assessment of the caregivers. Occupational therapists provided caregivers with problem-solving skills, environmental modifications and stress reduction training [31] to address caregivers’ needs at home. In all studies, both home sessions and telephone counselling were conducted [28,29,30,31]. The number of home visits differed between studies. In two of them [30,31], nine home sessions and one telephone counselling per month were conducted. In contrast, in the remaining studies, the number of home visits was reduced to five [28] or six [29]. The duration of the sessions in all studies was ninety minutes (home sessions) and thirty minutes (telephone counselling). All programs were conducted exclusively by occupational therapists. Only two of them stated that they were treating a specific dementia: Alzheimer’s disease [28,30].

### 3.7. Home-Based Intervention: Advancing Caregiver Training (ACT)

ACT was the third most used occupational therapy intervention (*n* = 3) [34,35,37] in the included studies. ACT consisted of a typed ‘action plan’ which included the specific behaviour of the people with dementia, the treatment goal, possible triggers and four management strategies (adapting the physical environment, assistive devices, simplifying communication and tasks, involving patients in the activity) [34]. Caregivers were instructed in stress reduction and self-care techniques [34] as well as in daily living activities such as dressing, bathing, grooming and feeding [35,37] through role-playing and explanatory videos. The number of sessions carried out in each study were eleven [34], six [35] and one [37]. The duration of the sessions in two of them was 1 h and in others the duration was not stated [34]. ACT was carried out exclusively by an occupational therapy in only one study [37]; in the remaining studies [34,35] there were more professionals within the team, such as nurses and doctors or rehabilitators.

### 3.8. Home-Based Intervention: Other Household Interventions

Other non-specific home-based interventions were carried out in the included studies (*n* = 10) [14,24,25,27,33,38,39,40,41,43]. These non-specific interventions were varied and included strategies such as home-based environmental interventions [14,24,25,27,33], home-based occupational therapy [38,39,40,41] and a home-based missing incident prevention program [43]. In all of these interventions, the caregiver received home-based training and/or counselling by the occupational therapist, although they did not fall into one of the three specific types presented previously.

### 3.9. Other Interventions

In the remaining studies (*n* = 9) [26,42,44,45,47,48,49,52,53], the interventions were very varied and can be delivered both in the home and in other settings. These interventions were: structured stress management courses [26], active psychoeducation interventions [42], dementia care education and activity programming [44], memory-making programs [45], telehealth [47], structured interventions [48], psychoeducational interventions for caregivers [49], and community-based occupational therapy in dementia programs (COTiD) [52,53].

### 3.10. Variables of Study and Measurement Instruments

The most frequent variables of interest in the included studies were consequences of caring for the people with dementia on caregivers, particularly caregiver depression, anxiety and stress (Table S1). Fifteen articles [25,31,32,34,35,36,39,41,42,45,48,49,50,52,53] assessed these variables using instruments such as Center for Epidemiological Studies Depression Scale (CES-D) [25,31,32,34,35,36,52]; Cornell Scale for Depression (CSD) [39], Geriatric Depression Scale (GDS) [35]; Hospital Anxiety and Depression Scale (HADS) [41,53]; Beck Depression Inventory (BDI) [45]; and Brief Symptom Inventory (BSI) [49]. Specifically, caregiver perceived stress was measured using Neuropsychiatric Inventory-Questionnaire (NPI-Q) [42]; Neuropsychiatric Inventory (NPI) [50]; and Relative’s Stress Scale (RSS) [48].

Caregiver burden was the second most studied variable (Table S1) Twelve of the articles [26,28,30,34,36,40,41,42,43,44,49,50,51] measured caregiver burden using scales such as Zarit Burden Interview (ZBI) [40,41,42,43,44,50,51]; Revised Memory and Behavior Problems Checklist (RMBPC) [28,30]; Caregiver Burden Inventory (CBI) [49]; Zarit Burden Short Form [34,36]; and Carers’ Checklist [26].

Other characteristics associated with caregivers were assessed to a lesser extent (Table S1). Their quality of life was assessed in five articles [32,33,39,41,52] using the Quality of Life–Alzheimer Disease scale [32,33], the European Quality of Life (EuroQol) [41], the Dementia Quality of Life Instrument (DqOL) [39] and the Health Survey Questionnaire (HSQ) [52]. In two articles [37,47], caregivers’ confidence was assessed using Caregiver Confidence Scale (CCS) [37] and Caregiving Mastery Index (CMI) [47]. In another four articles [28,32,34,42], care skill was assessed using the Task Management Strategy Index [28,32,34] and Care Skill Inventory (CSI) [42]. Caregiver well-being was measured in three articles [28,33,34] using the Perceived Change Index (PCI). Caregiver vigilance was assessed in one study [46] using the Caregiver Vigilance Scale (CVS). Finally, the caregiver’s sense of competence was measured by Sense of Competence Questionnaire (SCQ) [38,53] and, two previously cited measurement instruments, Task Management Strategy Index and RMBPC, were used to assess other variables such as caregiver discomfort and memory-related behaviours, respectively.

### 3.11. Main Limitations of Included Studies

In Table S3, we show the main limitations reported in the included studies. Those reported by the authors of included articles were small sample size (*n* = 12) [24,26,36,37,41,42,43,45,46,49,50,51], a lack of a control group or the presence of a control group which did not receive any form of intervention (*n* = 5) [32,34,41,43,45], a short duration of the intervention (*n* = 3) [35,49,52], low generalizability of results (*n* = 3) [37,50,53], losses during follow-up (*n* = 2) [24,48] and the lack of double blinding (*n* = 2) [38,39]. In the case of the remaining studies, the limitations were not homogeneous and were specific to each investigation.

## 4. Discussion

We explored the scientific evidence available in several databases and journals to de-scribe occupational therapy interventions for caregivers of dementia patients by carrying out a qualitative analysis of 31 articles with different types of occupational therapy interventions. We then classified these occupational therapy interventions into two categories, home-based occupational therapy interventions and other occupational therapy interventions. In the first category, we found three specific techniques for caregivers of dementia patients: TAP, ESP and ACT. Of these three techniques, TAP was the most frequently used occupational therapy intervention in the included studies.

In this scoping review, most of the included articles (*n* = 15) [14,24,25,26,27,28,29,30,31,32,33,34,35,36,37] were conducted in the United States such as Pennsylvania or Detroit, while only eight [38,39,40,41,48,49,52,53] were conducted in Europe. One reason for this is that the United States is one of the countries with higher research productivity in OT [54]. We should also underline that more than half of the included studies (*n* = 16) [33,34,35,36,37,40,41,42,43,44,46,47,50,51,52,53] were published in the last decade. This suggests that there has been an increasing number of publications related to occupational therapy interventions for dementia care and caregivers in recent years. The oldest included article in this review was published in 1991 [24], which indicates that while occupational therapists working with caregivers is relatively new, it has several years of evolution and scientific trajectory.

OT interventions were mainly focused on caregivers of people with Alzheimer’s disease. This result is in line with the current global situation of dementia. Alzheimer’s disease is the most common type of dementia, representing 60–70% of dementia cases [55] and affects between 5 and 8% of people over 60 worldwide [55]. Consequently, the number of caregivers providing care for people with Alzheimer’s disease is also very high. In the U.S, for example, almost half of all caregivers (48%) of older adults provide care for people with Alzheimer’s disease or another type of dementia [56].

Occupational therapy interventions for caregivers of people with dementia are mainly carried out at the homes of people with dementia. This is because the home is usually the context in which care is provided for the greatest amount of time [57]. Furthermore, dementia patients eventually become dependent on another person, usually a relative, and need help performing daily life activities, which are normally carried out at home [58]. Daily life activities are usually addressed by an Occupational Therapist or by trained caregivers [38]. Homes are the main setting for dementia patients and their caregivers during the early and middle stages of the disease, because in the severe stages most patients are transferred to nursing homes [59,60] where there are fewer opportunities to carry out occupational therapy interventions for caregivers.

Home-based occupational therapy interventions are the most used for caregivers of people with dementia. Occupational therapy is a discipline which aims to improve the fit between occupation, the people’s with dementia capabilities, and the environment in which they live in order to optimise their participation in meaningful activities [61]. Specifically, the TAP approach was the most used home-based occupational therapy intervention, possibly because its use in dementia from occupational therapy is supported by a significant quantity of scientific evidence [62,63]. Briefly, TAP is a person–environment–occupation framework with which dementia caregivers learn how to man-age behavioural symptoms through activities tailored to the remaining abilities of the PwD [64]. TAP has been shown to be effective in reducing both the amount of time caregivers need to provide care [32] and the number of neuropsychological symptoms (NPS) of the PwD’s [36], and it could help reduce healthcare costs [65].

The main health aspects associated to caregiving addressed by the occupational therapy interventions described in the included studies were behavioural symptoms (depression, anxiety, stress) and caregiver burden. One reason that could explain this is that balancing care for people with dementia with both their daily activities and the caregiver’s own activities can be very stressful [66]. In this regard, caregivers often experience daily caregiving challenges that result in an increased risk of depression and poor quality of life [67]. Another reason that may explain why it is the caregiver’s behavioural aspects that are most affected and therefore most studied in the included articles is the existence of NPS in the people with dementia. NPS are very common in PwD [68] and are associated with an increased level of stress caregivers [69], which could lead to an increased burden [70].

The results of this scoping review can be used to facilitate evidence-based occupational therapy intervention. It could be considered as a help document in which occupational therapists can consult which are the most commonly used interventions in caregivers of people with dementia and be able to design their interventions according to the characteristics described in this paper. We would like to highlight that in occupational therapy interventions by supporting the caregiver the care recipient is supported. To our knowledge, this suggests that we should include the person with dementia in the interventions, or at least take into account that with the intervention on the caregiver, we are surely influencing the quality of care for the person with dementia and therefore, their quality of life.

### Limitations

This scoping review has a number of limitations, which may influence the results obtained. We only included articles written in English or Spanish and with full-text available, which could lead to the loss of some important articles in this review. In addition, we performed the bibliographic search in only four databases, and although these databases are highly recommended [20], we did not include OTseeker or CINAHL databases and we might have missed some important articles. We did not assess the quality of the included studies because it is not mandatory in scoping reviews, so it is possible that some of the included studies have a low methodological quality. Finally, not all of the included articles measure the same variables or use the same measurement instruments, which complicates the comparison of the results between studies.

However, this scoping review presents a series of strengths. This research is a novel study since no other review has previously described the most used occupational therapy interventions for caregivers of people with dementia. Moreover, we would like to point out the large number of articles analysed (*n* = 31) in this scoping review, the majority of which (*n* = 19) were randomised clinical trials, a study design that provides a powerful response to questions of causality. Finally, this scoping review highlights a number of gaps in present knowledge: (i) most of the included studies are conducted in the United States, with only a minority conducted in Europe, none of which took place in Spain; (ii) most of the studies do not evaluate the results of the intervention in the long term, so more long-term intervention studies are needed; (iii) more studies are needed based on occupational therapy for caregivers of people with dementia.

## 5. Conclusions

Occupational therapy interventions for dementia caregivers are mainly home-based and carried out in caregivers of people with Alzheimer’s Disease, with the aim of reducing the caregivers’ depression, stress and anxiety. Three specific occupational therapy home-based interventions stand out: TAP, ESP and ACT. All of these interventions were carried out in weekly sessions which lasted between 1 to 1.5 h. TAP and ESP were carried out exclusively by an occupational therapist while ACT was provided by a multidisciplinary team which included an occupational therapist. This scoping review could be a useful tool for occupational therapists when designing base-evidence interventions for dementia caregivers.

## Figures and Tables

**Table 1:** Search strategies and databases.

Database	Search Strategy
PubMed	((alzheimer OR dementia OR parkinson) AND caregiver AND “occupational therapy”)
Scopus	((alzheimer OR dementia OR parkinson) AND caregiver AND “occupational therapy”)
EMBASE	(alzheimer OR ‘dementia’/exp OR dementia OR parkinson) AND (‘caregiver’/exp OR caregiver) AND (‘occupational therapy’/exp OR ‘occupational therapy’)
WOS	((alzheimer OR dementia OR parkinson) AND caregiver AND “occupational therapy”)
AJOT	(alzheimer OR dementia OR parkinson) AND caregiver AND “occupational therapy”
JOR	(alzheimer OR dementia OR parkinson) AND caregiver AND “occupational therapy”
POTP	[All: alzheimer] OR [All: dementia] OR [All: parkinson] AND [All: caregiver] AND [All: “occupational therapy”] AND [in Journal: Physical & Occupational Therapy In Pediatrics]
OTJR	(alzheimer OR dementia OR parkinson) AND caregiver AND “occupational therapy”
SJOT	[[All: alzheimer] OR [All: dementia] OR [All: parkinson]] AND [All: caregiver] AND [All: “occupational therapy”] AND [in Journal: Scandinavian Journal of Occupational Therapy]
AOTJ	[[All: alzheimer] OR [All: dementia] OR [All: parkinson]] AND [All: caregiver] AND [All: “occupational therapy”]
CJOT	[[All alzheimer] OR [All dementia] OR [All parkinson]] AND [All caregiver] AND [All “occupational therapy”] within Canadian Journal of Occupational Therapy
BJOT	[[All alzheimer] OR [All dementia] OR [All parkinson]] AND [All caregiver] AND [All “occupational therapy”] within British Journal of Occupational Therapy
OTI	dementia and alzheimer and parkinson and caregiver and occupational therapy
HKJOT	[[All alzheimer] OR [All dementia] OR [All parkinson]] AND [All caregiver] AND [All “occupational therapy”] within Hong Kong Journal of Occupational

AJOT: American Journal of Occupational Therapy; AOTJ: Australian Occupational Therapy Journal; BJOT: British Journal of Occupational Therapy; CJOT: Canadian Journal of Occupational therapy; HKJOT: Hong Kong Journal of Occupational therapy; JOR: Journal of Occupational Rehabilitation; OTJR: Occupation, Participation, and Health; OTI: Occupational Therapy International; POTP: Physical & Occupational Therapy In Pediatrics; SJOT: Scandinavian Journal of Occupational Therapy; WOS: Web of Science.

## Data Availability

The data presented in this study are available on request from the corresponding author.

## Supplementary Materials

The following supporting information can be downloaded at: https://www.mdpi.com/article/10.3390/healthcare10091764/s1, Table S1: Main characteristics of the studies included in this scoping review; Table S2: Main characteristics of the OT interventions in PwD carried out in the included studies; Table S3: Limitations, funding and conflicts of interest declared in the included studies.
