# Supplementary material for: Occupational Therapy Interventions for Dementia Caregivers: Scoping Review
Source: Healthcare (Basel). 2022 Sep 14;10(9):1764. doi: 10.3390/healthcare10091764 (PMC9498417; doi:10.3390/healthcare10091764)
Supplement: Supplementary file 1 [file healthcare-10-01764-s001.zip › healthcare-1852066-supplementary.pdf]

Table S1. Main characteristics of the studies included in this scoping review

Author., year	Design	Sample (n), country	Participants	Intervention/ Comparator	Evaluation	Study outcomes
Pynoss J & Russell J., 1991 [24]	Pilot	25, United States. Loss to follow-up (n=13)	12 people with AD-caregiver dyads. Average age of caregivers 70.8y. Average care time 40.1m.	Home environmental intervention/NA	Pre-and post-evaluation.	Effectiveness of home intervention assessed by Home Environmental Checklist. Caregiver-experienced problems assessed by Problems and barriers survey.
Gitlin L et al., 1999 [25]	Quasi	100, United States. Loss to follow-up (NS)	100 caregivers of PwD. Average age of caregivers 59y. Average care time 42m.	Home environmental interventions/NA	Pre-and post-evaluation.	Intervention adherence assessed by IADLs and CES-D
Mitchell E., 2000 [26]	Quasi	7, United States. Loss to follow-up (n=1)	6 caregivers of PwD. Average age of caregivers NS. Average care time NS.	Structured stress management course/NA	Pre- and post-evaluation	Caregiver burden assessed by the Carers' Checklist.
Corcoran MA & Gitlin L., 2001 [27]	nRCT	100, United States. Loss to follow-up (NS)	100 caregivers of PwD. Average age of caregivers 59.3y. Average care time 41.5m.	Home environmental intervention/NA	Pre- and post-evaluation.	Patients' behavioural problems assessed by the RMBPC.
Gitlin L et al., 2001 [14]	RCT	202, United States. Loss to follow-up (n=31)	171 caregivers of people with AD. Average age of caregivers 61y. Average care time 45m.	Home environmental intervention/Education materials and a booklet	Pre- and post-evaluation	Performance of the PwD assessed by the MBPC and the FIM. Caregiver self-efficacy assessed by the approach of Haley and colleagues. Caregivers upset assessed by a 5-point scale.
Gitlin L et al, 2003 [28]	RCT	255, United States. Loss to follow-up (n=65)	190 caregivers of people with AD. Average age of caregivers 60.5y. Average care time 4.3y.	ESP/Usual Care	Pre-, post-evaluation and 6-, 12-, 18-m follow-up	Caregiver burden assessed by the RMBPC. Level of ADL assistance required of the PwD assessed by the FIM. Caregiver perceived mastery assessed by the CMI. Caregiver skill enhancement assessed by the TSMI. Caregiver well-being assessed by the PCI.

Brodaty H & Low LF., 2004 [45]	Quasi	24, Australia.<br>Loss to follow-up (NS)	24 PwD-caregiver dyads.<br>Average age of caregivers 67y.<br>Average care time NS.	The Making Memories program/NA	Pre-, post-evaluation and 24-w follow-up	Patient Memory and behaviour assessed by the RMBPC.<br>Patient cognitive functioning assessed by the MMSE.<br>Participants quality of life assessed by the EQLS<br>Participants psychologic status assessed by the GHQ-12.<br>Caregiver depression assessed by BDI.
Nobili A. et al., 2004 [48]	Pilot	69, Italy.<br>Loss to follow-up (n=30)	39 PwD-caregiver dyads.<br>Average age of caregivers 56y.<br>Average care time 3y.	Structured intervention/the counselling program of the Federazione Alzheimer Italia	Pre-, post-evaluation and 12-m follow-up	Caregiver stress assessed by the RSS.<br>Patients' problem behaviors assessed by the SBI-C.<br>Patients' functionality assessed by ADL and IADL scales.
Gitlin L et al., 2005 [29]	RCT	190, United States.<br>Loss to follow-up (n=63)	127 caregivers of PwD.<br>Average age of caregivers 60.8y.<br>Average care time 4.3y.	ESP/Usual care	Pre-, post-evaluation and 12-m follow-up.	Caregiver memory-related behaviors assessed by RMBPC<br>Caregiver upset assessed by the TMSI.
Graff M et al., 2006 [38]	RCT	135, The Netherlands.<br>Loss to follow-up (n=30)	105 PwD-caregiver dyads.<br>Average age of caregivers 63.7y.<br>Average care time NS.	OT at Home/No OT.	Pre-, post-evaluation and 6-,12-m follow-up.	Patients' daily functioning assessed by the AMPS and the IDDD.<br>Caregivers' sense of competence assessed by the SCQ.
Graff M et al., 2007 [39]	RCT	135, The Netherlands.<br>Loss to follow-up (n=30)	105 PwD-caregiver dyads.<br>Average age of caregivers 63.7y.<br>Average care time NS.	OT at Home/No OT.	Pre-, post-evaluation and 6-,12-m follow-up.	Quality of life assessed by the DQOL.<br>Health status assessed by the GHQ-12.<br>Mood assessed by the CSDD.
Onor ML et al., 2007 [49]	RCT	32, Italy.<br>Loss to follow-up (n=0)	32 people with AD-caregiver dyads.<br>Average age of caregivers 63.7y.<br>Average care time NS.	ROT for patients and Psychoeducational intervention for caregivers / No form of intervention	Pre-, 2- m and post-evaluation.	Caregivers' mood assessed by the BSI.<br>Caregivers' burden assessed by the CBI.<br>Patients' cognitive function assessed by the MODA and the MMSE.<br>Patients' mood assessed by the GDS.<br>Patients' functionality assessed by the IADL and ADL.
Hilgeman M et al., 2007 [30]	RCT	253, United States.<br>Loss to follow-up (n=10)	243 caregivers of people with AD.<br>Average age of caregivers 60.9y.<br>Average care time 4.3y.	ESP/usual care	Pre-, 6-m and post-evaluation.	Caregiver depression assessed by the CES–D<br>Caregiver burden assessed by the RMBPC.

Chee KY et al.,2007 [31]	RCT	129, United States. Loss to follow-up (n=24)	105 caregivers of PwD. Average age of caregivers 60.9y. Average care time NS.	ESP/usual care	Pre-, 6-m and post-evaluation and 18-m follow-up.	Caregiver depression assessed by the CES-D
Gitlin L et al., 2008 [32]	Pilot	60, United States. Loss to follow-up (n=4)	56 PwD-caregiver dyads. Average age of caregivers 65y. Average care time NS.	TAP/usual care	Pre- and post-evaluation.	Caregiver depression assessed by the CES-D. Caregiver skill enhancement assessed by the TMSI. Caregiver perceptions of life quality in dementia patients assessed by the QoL-AD Patient behaviour assessed by the ABID and the RMBPC. Patient depression assessed by the CSDD.
Gitlin L et al., 2010 [33]	RCT	284, United States. Loss to follow-up (n=75)	209 PwD-caregiver dyads. Average age of caregivers 62.2y. Average care time 4y.	Environmental intervention/usual care	Pre- and post-evaluation and 9-m follow-up.	Patient functional independence assessed by the FIM. Caregiver perceptions of life quality in dementia patients assessed by the QoL-AD. Caregiver well-being assessed by the PCI.
Gitlin L. et al., 2010 [34]	RCT	272, United States. Loss to follow-up (n=55)	272 caregivers of PwD. Average age of caregivers 21y. Average care time NS.	ACT/Usual care	Pre- and post-evaluation and 16-, 24-w follow-up.	Caregiver´s burden assessed by the ZBI. Caregiver´s depressive symptoms assessed by the CES-D. Caregiver´s well being assessed by the PCI. Caregiver´s skill enhancement assessed by the TMSI.
Voigt-Radloff S et al., 2011 [52]	RCT	141, Germany. Loss to follow-up (n=12)	129 people with AD-caregiver dyads. Average age of caregivers 64.7y. Average care time NS.	COTiD/COTC	Pre- and post-evaluation and 16- and 26-w follow-up.	Patient’s daily functioning assessed by the IDDD Caregiver’s mood assessed by the CES-D. Patient’s quality of life assessed by the DQOL Caregiver’s quality of life assessed by the SF-12.

Sturkenboom I et al., 2013 [40]	RCT	43, The Netherlands. Loss to follow-up (n=3)	40 people with PD-caregiver dyads. Average age of caregivers 62y. Average care time NS.	Home-based OT/No OT	Pre- and post-evaluation and 3-m follow-up.	Caregiver's burden assessed by the ZBI. Patient's functionality assessed by the AMPS Patient's perceived performance assessed by the COPM.
DiZazzo-Miller R et al., 2014 [35]	nRCT	72, United States. Loss to follow-up (n=19)	53 caregivers of people with AD. Average age of caregivers 60y. Average care time NS.	FCTP/NA	Pre- and post-evaluation and 3-m follow-up.	Caregiver's depression assessed by the GDS.
Sturkenboom I et al., 2014 [41]	RCT	180, The Netherlands. Loss to follow-up (n=6)	174 caregivers of people with PD. Average age of caregivers 66y. Average care time NS.	Home-based OT/No OT	Pre- and post-evaluation and 6-m follow-up.	Caregiver's burden assessed by the ZBI. Caregiver's proactive coping skills assessed by the UPCC. Caregiver's mood assessed by the HADS. Caregiver's quality of life assessed by the EQ-5D.
Gitlin L et al., 2018 [36]	RCT	164, United States. Loss to follow-up (n=4)	160 veterans with dementia-caregiver dyads. Average age of caregivers 72.4y. Average care time NS.	TAP involving veterans with dementia/ telephone-based dementia education	Pre- and post-evaluation and 8-m follow-up.	Patient's behavioural symptoms assessed by the NPI-C. Patient's functional dependence assessed by the CAFU. Patient's pain assessed by the Pain Intensity Scale. Caregiver's depression assessed by the CES-D. Caregiver's burden assessed by the ZBI-12.
O'Connor C et al., 2019 [46]	Pilot	20, Australia. Loss to follow-up (n=0)	20 people with FTD-caregiver dyads Average age of caregivers 62.5y. Average care time NS.	TAP/telephone-based dementia education	Pre- and post-evaluation	Patient's general cognition assessed by the MoCA. Patient's behavioural symptoms assessed by the NPI-C. Patient's functional dependence assessed by the DAD. Patient's quality of life assessed by the EQ-5D. Caregivers vigilance assessed by the CSV.

De Oliveira A et al., 2019 [51]	Pilot	21, Brazil. Loss to follow-up (n=0)	21 PwD-caregiver dyads. Average age of caregivers 58.7y. Average care time NS.	TAP–outpatient version/Psychoeducation intervention	Pre- and post-evaluation	Patient's neuropsychiatric symptoms assessed by the NPI-C. Caregiver burden assessed by the ZBI.
Tang SH et al., 2018 [42]	RCT	43, China. Loss to follow-up (n=5)	38 PwD-caregiver dyads. Average age of caregivers 57.1y. Average care time NS.	Active psychoeducation /Passive psychoeducation	Pre- and post-evaluation and 3-m follow-up	Caregiver's skills and competence assessed by the CSI. Caregiver's burden assessed by the ZBI. Caregiver's distress assessed by the NPI-Q.
Lau WM et al., 2019 [43]	nRCT	54, China. Loss to follow-up (n=10)	44 PwD-caregiver dyads. Average age of caregivers NS. Average care time NS.	Home-based missing incident prevention program/NA	Pre- and post-evaluation and 3- and 12-m follow-up	Patient's cognition assessed by the MoCA. Patient's functionality assessed by the DAD. Patient's neuropsychiatric symptoms assessed by the NPI-Q Patient's risk of wandering assessed by the WRS. Caregiver's burden assessed by the ZBI.
Novelli M et al., 2018 [50]	Pilot	30, Brazil. Loss to follow-up (n=0)	30 PwD-caregiver dyads. Average age of caregivers 66y. Average care time NS.	TAP-Brazilian version/usual care	Pre- and post-evaluation	Patient's behavioural symptoms assessed by the NPI-Q. Patient's quality of life assessed by the QoL-AD Caregiver's distress assessed by the NPI-C. Caregiver's burden assessed by the ZBI.
Lai FH et al., 2020 [44]	RCT	106, China. Loss to follow-up (n=6)	100 PwD-spouse caregiver dyads. Average age of caregivers NS. Average care time NS.	Dementia care education+Activity scheduling/ Dementia Care Education	Pre- and post-evaluation	Caregiver's burden assessed by the ZBI. Patient's behavioural symptoms assessed by the RMBPC.
Laver K et al., 2020 [47]	RCT	63, Australia. Loss to follow-up (n=10)	53 PwD- caregiver dyads. Average age of caregivers 70y. Average care time 3y.	Telehealth/Home visits	Pre- and post-evaluation	Caregiver's confidence assessed by the CMI. Caregiver's care perception assessed by the PCI.
DiZazzo-Miller R et al., 2020 [37]	RCT	36, United States. Loss to follow-up (n=0)	36 caregivers of people with AD. Average age of caregivers 50y. Average care time 3y.	The FCTP focused on ADL/ The FCTP focused on standard care	Pre-and post-evaluation and 3-m follow- up	Caregiver's confidence assessed by the Caregiver Confidence Scale.

Wenborn J et al., 2021 [53]	RCT	468, United Kingdom. Loss to follow-up (n=122)	346 PwD-caregiver dyads. Average age of caregivers 69.1y. Average care time NS.	COTiD UK version/Usual care	Pre-and post-evaluation and 12-, 26-, and 52-w follow- up	Caregivers' sense of competence assessed by the SCQ. Caregiver's mood assessed by the HADS.

ABID, Agitated Behaviors in Dementia Scale; ACT, Advancing Caregiver Training; ADL, Activities of Daily Living; AD, Alzheimer's disease; AMPS, Assessment of motor and process skills; BDI, Beck Depression Inventory; BSI, Brief Symptom Inventory; CAFU, Caregiver Assessment of Function and Upset Scale; CBI, Caregiver Burden Inventory; CES-D, Center for Epidemiologic Depression Scale; CMI, Caregiving Mastery Index; COPM, Canadian Occupational Performance Measure; COTiD, Community Occupational Therapy in Dementia Program; COTC, Community Occupational Therapy Consultation; CSDD, Cornell Scale for Depression in Dementia; CSI, Care Skill Inventory; CVS, Caregiver Vigilance Scale; DAD, Disability Assessment for Dementia; DQOL, Dementia Quality of Life Instrument; EQ-5D, EuroQol-5D; EQLS, European Quality of Life survey; ESP, Environmental Skill-Building Program; FCTP, Family Caregiver Training Program; FIM, Functional Independence Measure; FTD, Frontotemporal dementia; FTD-FRS, Frontotemporal Dementia Rating Scale; GDS, Geriatric Depression Scale; GHQ-12, General Health Questionnaire; HADS, Hospital Anxiety and Depression Scale; IADL: Instrumental Activities of Daily Living; IDDD, Interview of Deterioration in Daily activities in Dementia; MBPC, Memory and Behavior Problems Checklist; MMSE, Mini-Mental State Examination; m, months; MoCA, Montreal Cognitive Assessment; MODA, Milan Overall Dementia Assessment; NA, Not applicable; NPI, Neuropsychiatric Inventory; NPI-C, Neuropsychiatric Inventory-Clinician rating scale; NPI-Q, Neuropsychiatric Inventory-Questionnaire; nRCT, non-randomized controlled trial; NS, Not stated; OT, Occupational Therapy; PCI, Perceived Change Index; PD, Parkinson's disease; Pilot, Pilot study; PwD, people with dementia; QoL-AD: Quality of Life-AD Scale; Quasi, Quasi-experimental study; RCT, randomized controlled trial; RMBPC, Revised Memory and Behavior Problems Checklist; RSS, Relative's Stress Scale; ROT, Reality Orientation Therapy; SBI-C, Spontaneous Behavior Interview; SCQ, Sense of Competence Questionnaire; SF-12,12-Item Short-Form Health Survey; TAP, Tailored Activity Program; TMSI, Task Management Strategy Index; UPCC, Utrecht Proactive Coping Competence Scale; w, weeks; WRS, Wander Risk Scale; ZBI, Zarit Burden Interview; ZBI-12, Short Form Zarit Burden Interview; y, years.

Table S2. Main characteristics of the OT interventions in PwD carried out in the included studies.

**Authors., year**	**Participants (n) and diagnostic**	**Intervention activities**	**Duration (w)**	**Sessions**	**Intervention manager**	**Main results**
Pynoss J & Russell J., 1991 [24]	12 people with AD-caregiver dyads.	IG: Home environmental intervention. The caregiver identifies the barriers and problems of the PwD in performing ADL at home. Then, the occupational therapist trains him/her on how to address these barriers.<br>CG: NA.	NS	NS	OTs, clinical gerontologist, psychologist	66% of the problems addressed were evaluated by the caregivers as having been effective.<br>89% of the initially effective continued to be effective at the follow-up.
Gitlin L et al., 1999 [25]	100 caregivers of PwD.	IG: Home environmental intervention. OTs worked with the caregiver to identify problems and develop environmental strategies. Caregivers were instructed about recommended strategies.<br>CG: NA.	12	5 sessions.<br>One-weekly 90-minute session.	OTs	Caregivers of PwD with high IADL functioning were more likely to adhere to treatment.
Mitchell E., 2000 [26]	6 caregivers of PwD.	IG: Structured stress management course. A 4-weeks course for caregivers, which included content about stress, anxiety, problems solving and assertiveness and strategies such as role playing, videos and relaxation.<br>CG: NA.	4	4 sessions.<br>Duration NS.	OTs	The burden of care scales overall reduced while the caregivers showed a raised awareness in their questionnaire ratings.
Corcoran MA & Gitlin L., 2001 [27]	100 caregivers of PwD.	IG: Home environmental intervention. Caregivers identify up to three problem areas to address. To resolve each problem, the OTs and caregiver designed environmental strategies. Methods such as role-play, paper and pencil exercises, discussion, demonstration, practice, and simulation were used.<br>CG: NA.	8	5 sessions.<br>Two-monthly 90-minute sessions.	OTs	Caregiver-centered concerns, catastrophic reactions, wandering and incontinence were the most frequently identified problems by caregivers.<br>84% of caregivers used strategies that modified the task.<br>83% of caregivers used strategies that modify social environments.<br>74% of caregivers used strategies that modified objects of the environment.

Gitlin L et al., 2001 [14]	171 caregivers of people with AD.	IG: Home environmental intervention. Intervention aimed at helping caregivers simplify objects in the home, break down tasks, and involve other members of the family network or formal supports in daily caregiving tasks. Methods such as role-play, direct observation, and interviewing were used.<br>CG: Participants received education materials and a booklet describing home environmental safety tips at the conclusion of the study.	12	5 sessions.<br>Two-monthly 90-minute sessions.	OTs	Intervention spouses reported reduced upset (p=0.049).<br>Women caregivers reported enhanced self-efficacy in managing behaviors (p=0.038).<br>Women caregivers reported enhanced self-efficacy in managing functional dependency (p=0.049).
Gitlin L et al, 2003 [28]	190 caregivers of people with AD.	IG: ESP. The program consisted of four components: education about dementia and the impact of the home environment on troublesome behaviors; instruction in problem solving; implementation of environmental strategies; and generalization of strategies to emerging problems.<br>CG: Usual care. CG received resource information a each testing occasion	24	5 sessions.<br>One-monthly 90-minute session.	OTs	Compared with controls, IG caregivers reported less upset with memory-related behaviors (p=0.027) and better affect (p=0.034).
Brodaty H & Low LF., 2004 [45]	24 PwD-caregiver dyads.	IG: The Making Memories program. This program comprised: discussion, PwD participated in a memory loss group and caregivers in an education and support group; and behavioral modification, which consisted of weekly leisure activity groups.<br>CG: NA	18	18 sessions.<br>Two-weekly 60-minute sessions.	OTs	Patients' psychological morbidity decreased over time (p=0.005).<br>Caregivers' quality of life was high after the program (p=0.042).<br>Caregivers' distress was less after the program (p=0.007).
Nobili A. et al., 2004 [48]	39 PwD-caregiver dyads.	IG: Structured intervention. The OTs gave practical advice on strategies to prevent and manage problem behavior, to maintain the PwD's residual functional abilities and to modify home barriers.<br>CG: Counseling program of the Federazione Alzheimer Italia. It includes free help line, information about the rights of PwD and specialized clinical centers.	2	2 sessions.<br>Two-weekly 60-90-minute sessions.	OTs, psychologist	Compared with controls, IG caregivers presented a lower mean problem behavior score (p<0.03).<br>The main determinant of institutionalization seemed to be the level of caregiver stress (p = 0.03).
Gitlin L et al., 2005 [29]	127 caregivers of PwD.	IG: ESP. Six OT sessions to help families modify the environment to support daily function of the PwD and reduce caregiver burden.<br>CG: Usual care.	24	6 sessions.<br>Five-monthly 90-minute home visits and one-monthly telephone session.	OTs	At 6 months, compared with controls, IG caregivers reported improved skills (p=0.028), less need for help in providing assistance (p=0.043), and fewer behavioural occurrences (p=0.019).

						At 12 months, compared with controls, IG caregivers' affect improved (p=0.033).
Graff M et al., 2006 [38]	105 PwD-caregiver dyads.	IG: OT at home. PwD and primary caregivers learnt to choose meaningful activities. Then, caregivers were trained to use effective supervision, problem solving, and coping strategies to sustain the patients' and their own autonomy and social participation.<br>CG: Received OT after completion of the study.	5	10 sessions.<br>Two-weekly 60-minute sessions.	OTs	At 6 weeks, compared with controls, IG caregivers felt more competent (p<0.0001).<br>At week 12, the significant results found were maintained.
Graff M et al., 2007 [39]	105 PwD-caregiver dyads.	IG: OT at home. PwD and primary caregivers learnt to choose meaningful activities. Then, caregivers were trained to use effective supervision, problem solving, and coping strategies to sustain the patients' and their own autonomy and social participation.<br>CG: Received OT after completion of the study.	5	10 sessions.<br>Two-weekly 60-minute sessions.	OTs	At 6 weeks, compared with controls, both IG patients and caregivers improved their quality of life (p<0.0001), health status (p<0.0001) and mood (p<0.0001).<br>At week 12, the significant results found were maintained.
Onor ML et al., 2007 [49]	32 people with AD-caregiver dyads.	IG: ROT for PwD and psychoeducational intervention for caregivers. Caregivers received information about dementia and were taught to repeat some of the activities used in ROT. This intervention, addressed the feelings experienced by the caregivers.<br>CG: Patients received acetylcholinesterase inhibitors and caregivers received no intervention.	16	16 sessions.<br>One-weekly 60-minute session.	OTs, psychiatrist, psychologist, educator	There was a difference in anxiety (p=0.048) between pre- and post-evaluation in the IG. There was a difference in caregivers' burden between pre- and post-evaluation in both the IG (p=0.027) and CG (p=0.014).
Hilgeman M et al., 2007 [30]	243 caregivers of people with AD.	IG: ESP. OTs worked in the home with caregivers to tailor a program addressing the specific needs of the dyad by the implementation of problem-solving techniques for manipulating the environment.<br>CG: Usual care. Information and referral to services, which might resemble typical resources offered to Alzheimer's caregivers in the community.	48	9 sessions.<br>First six months: Five-monthly 90-minute home visits and one-monthly 30-minute telephone call.<br>Next six months: one-monthly 90-minute home visits and three-monthly 30-minute telephone call.	OTs	Caregivers low in positive aspects of caregiving showed the greatest benefit from intervention across 12 months (p=0.0038).
Chee KY et al.,2007 [31]	105 caregivers of PwD.	IG: ESP. OTs provided caregivers with problem-solving skills, environmental modifications, and stress reduction training.<br>CG: Usual care.	48	9 sessions.<br>First six months: Five-monthly 90-minute home	OTs	There was not an improvement of caregivers' psychological factors after the intervention (p=0.068).

				visits and one-monthly 30-minute telephone call. Next six months: one-monthly 90-minute home visits and three-monthly 30-minute telephone call.		
Gitlin L et al., 2008 [32]	56 PwD-caregiver dyads.	IG: TAP. Caregivers were instructed in stress-reducing techniques (deep breathing) to establish a calm emotional tone. As caregivers mastered activity use, interventionists generalized strategies to care problems and instructed them on how to downgrade activities for future declines.<br>CG: Usual care	16	8 sessions.<br>Six-monthly 90-minute home visits and two-monthly<br>15-minute telephone call.	OTs	At 4 months, compared with controls, IG caregivers reported a reduction of problem behaviors (p=0.009), fewer hours doing things for PwD (p=0.005), fewer hours on duty (p=0.001) and also reported greater mastery (p=0.013), and greater use of simplification techniques (p=0.023).
Gitlin L et al., 2010 [33]	209 PwD-caregiver dyads.	IG: Environmental intervention. OTs trained caregivers to modify home environments, daily activities, and communications to support PwD capabilities.<br>CG: Usual care. It included tips from the Alzheimer's Association and government agencies on home safety and managing patient behaviors, functional decline, and caregiver stress.	16	12 sessions.<br>Duration NS.	OTs, nurse, research staff	At 4 months, compared with controls, IG caregivers reported improvement in well-being (p=0.002) and confidence using activities (p=0.002).<br>At 9 months, compared with controls, IG caregivers reported an improvement in their lives overall (p<0.01), disease understanding (p=0.001) and confidence managing behaviors (p<0.01).
Gitlin L. et al., 2010 [34]	272 caregivers of PwD.	IG: ACT. The OTs brainstormed with caregivers to identify acceptable management strategies. A typed "action plan" was provided stating targeted behavior, treatment goal, potential triggers, and management strategies (adapting physical environment, assistive devices, simplifying communication and tasks, engaging patients in activity).<br>CG: Usual care. These participants did not receive any intervention contact.	24	11 sessions.<br>Duration NS.	OTs, nurse	At 4 months, compared with controls, IG caregivers reported an improvement in occurrence of the primary targeted problem behavior for ACT (p=0.002).<br>At 6 months compared with controls, IG caregivers reported an improvement in understanding the disease (p=0.001) and confidence managing behaviors (p=0.001).

Voigt-Radloff S et al., 2011 [52]	129 people with AD-caregiver dyads.	IG: COTiD. The OTs defined, together with the PwD and their caregiver, more effective compensatory and environmental strategies to adapt both the environment and some daily activities to the PwD.<br>CG: COTC. A semistructured consultation which included an explanation and a talk on individual problems that arose from the PwD's and caregiver's needs.	5	10 sessions.<br>Two-weekly 60-minute sessions.	OTs	Caregivers outcomes did not differ significantly between the IG and CG at post evaluation.
Sturkenboom I et al., 2013 [40]	40 people with PD-caregiver dyads.	IG: Home-based OT. The intervention provided caregivers with information about the impact of the disease on the patient's daily functioning, possible care resources and adaptations, and supervision skills training.<br>CG: No OT. Other medical, psychosocial or allied health care interventions rather than OT.	10	16 sessions.<br>Two-weekly 45-60-minute home sessions.	OTs	At 3 months, compared with controls, IG caregivers showed a small positive trend in caregiver burden but was not significant.
DiZazzo-Miller R et al., 2014 [35]	53 caregivers of people with AD.	IG: FCTP. It included three modules: 1. training caregivers on eating and feeding techniques; 2. focused on toileting and transferring, 3. covered bathing and dressing, including an overview of grooming. Training methods included presentations with short explanations, group discussions, and role-playing.<br>CG: NA.	3	6 sessions.<br>Two-weekly 60-minute sessions.	OTs, patient advocate, training specialist, rehabilitation administrator.	Caregivers showed a significant gain in knowledge of how to effectively assist with communication and nutrition ($p<0.001$); transfers and toileting, ($p<0.003$); and bathing and dressing ($p<0.01$) of their care recipients after intervention.
Sturkenboom I et al., 2014 [41]	174 caregivers of people with PD.	IG: Home-based OT. The intervention provided caregivers with information about the impact of the disease on the PwD's daily functioning, possible care resources and adaptations, and supervision skills training.<br>CG: Other medical, psychosocial or allied health care interventions rather than OT.	10	16 sessions.<br>Two-weekly 60-minute home sessions.	OTs	At 3 months, compared with controls, IG caregivers showed an improved quality of life ($p=0.006$).
Gitlin L et al., 2018 [36]	160 veterans with dementia-caregiver dyads.	IG: TAP. Caregivers were provided with activity prescriptions detailing activity goals, how to set up the environment to support activities and specific strategies for their implementation. Also, caregivers learned to use activities through demonstration with veterans.	16	8 sessions.<br>Two-monthly 60-minute sessions.	OTs	At 4 months, compared to controls, IG caregivers reported less behavior-related distress ($p=0.03$).

		CG: Telephone-based dementia education. A masters-level team member with experience educated the caregivers. Information was provided on relevant topics (home safety, dementia), with no discussion of activity or behavioural symptom.				
O'Connor C et al., 2019 [46]	20 people with FTD-caregiver dyads.	IG: TAP. Caregivers were educated about dementia and behaviors, learning skills in activity simplification and communication, and practicing ways to effectively engage the person with dementia in activities.<br>CG: Telephone-based dementia education. Caregivers received telephone calls based around a book on general dementia-related matters.	16	8 sessions.<br>Two-monthly 60-minute sessions.	OTs	IG caregivers did not improve statistically significantly more than CG caregivers.
De Oliveira A et al., 2019 [51]	21 PwD-caregiver dyads.	IG: TAP-outpatient version. This intervention included a caregiver's education about dementia symptoms and how to manage neuropsychiatric symptoms and learn stress reduction techniques.<br>CG: Psychoeducation intervention. Regular care anc psychoeducation group sessions.	12	8 sessions.<br>One-weekly 60-minute session.	OTs	Compared with controls, IG caregivers had a decrease in burden ($p=0.003$).
Tang SH et al., 2018 [42]	38 PwD-caregiver dyads.	IG: Active psychoeducation. The OTs designed activities tailored to the capacity of the PwD and assigned them as "a home-based program", then OTs and caregivers discussed in depth their experiences with the home-based program.<br>CG: Passive psychoeducation. A discussion based on dementia care education booklet.	NS	6 sessions.<br>60-minute sessions.<br>Frequency NS	OTs, nurse	Compared with controls, IG caregivers showed an improvement of caregiving skills ($p<0.001$), burden ($p<0.005$) and distress ($p<0.05$)
Lau WM et al., 2019 [43]	44 PwD-caregiver dyads.	IG: Home-based missing incident prevention program. Caregiver education about dementia, prescription of assistive devices, environmental modification, and redesigning of daily life routine tasks.<br>CG: NA.	NS	The number of sessions depended on the needs of the patients.	OTs, nurse	At 3 and 12 months, the caregivers' stress decreased ($p< 0.001$) and ($p<0.005$), respectively.
Novelli M et al., 2018 [50]	30 PwD-caregiver dyads.	IG: TAP-Brazilian version. This intervention focuses on matching activities to the cognitive and functional capabilities, previous roles, habits, and interests of the PwD, as well as training family caregivers in their use as part of daily care.	16	8 sessions.<br>Two-monthly 60-minute sessions.	OTs	At 4 months, compared with controls, IG caregivers reported a reduction in distress ($p<0.001$), and an improvement in quality o[illegible] life ($p=0.02$).

		CG: Usual care.				
Lai FH et al., 2020 [44]	100 PwD-spouse caregiver dyads.	IG: Dementia Care Education + activity scheduling. This is a caregiver-delivered intervention in which they chose things that PwD had been avoiding, in order to be addressed.<br>CG: Dementia care education. Weekly themed topics, such as the importance of exercise or sleep management were taught.	12	13 sessions.<br>One-weekly session.<br>Duration NS	OTs	Compared with controls, IG caregivers had a significant reduction on burden (p< 0.001)
Laver K et al., 2020 [47]	53 PwD- caregiver dyads.	IG: Telehealth intervention. Two consultations in the home with the OTs and the remaining sessions were provided using telehealth technologies. The sessions included environmental assessment and rapport building.<br>CG: Home visits. The OTs worked with the caregiver to problem solve, educate, and build skills.	16	8 sessions.<br>Two-monthly 60-minute sessions.	OTs, nurse	Both groups reported significant improvements in perceptions of caring but there were no statistically significant differences between groups (p=0.09)
DiZazzo-Miller R et al., 2020 [37]	36 caregivers of people with AD.	IG: FCTP focused on ADL. PowerPoint presentation covering the three activities of daily living ADL modules. Each module was followed by a hands-on demonstration session.<br>CG: FCTP focused on standard care. PowerPoint presentation on standard care consisted of general information on the different types of dementia and helpful resources online.	NS	1 session.<br>60-minute session.	OTs	Caregivers in the IG and CG experienced a significant increased confidence after intervention.
Wenborn J et al., 2021 [53]	346 PwD-caregiver dyads	IG: COTiD. First, the OTs assessed the two participants and home environment. Second, the OTs defined, together with the PwD and their caregiver, their lifestyle goals. This intervention can be carried out at PwD's home or at a community setting.<br>CG: Usual care which consisted in standard occupational therapy.	10	10 sessions.<br>One-weekly 60-minute session.	OTs	Caregivers' sense of competence and depression scores did not differ between groups at post-evaluation.

ACT, Advancing Caregiver Training; ADL, Activities of Daily Living; AD, Alzheimer's disease; CG, Control Group; COTiD, Community Occupational Therapy in Dementia Program; COTC, Community Occupational Therapy Consultation; ESP, Environmental Skill-Building Program; FCTP, Family Caregiver Training Program; FTD, Frontotemporal dementia; IADL: Instrumental Activities of Daily Living; IG, Intervention Group; NA, Not applicable; NS, Not stated; OT, Occupational Therapy; OTs, Occupational Therapist; PD, Parkinson's disease; PwD, people with dementia; ROT, Reality Orientation Therapy; TAP, Tailored Activity Program.

Table S3. Limitations, funding and conflicts of interest declared in the included studies.

Author., year	Main limitations	Funding sources	Conflicts of interest
Pynoss J & Russell J., 1991 [24]	Small sample size, losses of follow-up.	The Andrus Foundation of the American Association of Retired Persons and the Alzheimer's Disease Program of the California Department of Health Services and the Tingstad Alzheimer's Disease Research Fund.	No competing interests
Gitlin L et al., 1999 [25]	Unable to obtain ratings of acceptance of strategies that were independent of the interventionist.	The National Institute on Aging.	No competing interests
Mitchell E., 2000 [26]	Small sample size.	The Mental Health Foundation.	No competing interests
Corcoran MA & Gitlin L., 2001 [27]	Unable to determine inter-rater agreement among therapists.	The National Institute on Aging.	No competing interests
Gitlin L et al., 2001 [14]	Intervention effects were examined at one time point immediately following completion of the intervention.	The National Institute on Aging.	No competing interests
Gitlin L et al, 2003 [28]	Use of multiple end points, resulting in three statistically significant main effects.<br>Unclear whether the positive change that occurred in the IG is due to social attention by a health professional or the environmental content of the intervention given that their comparison was a usual care group (CG).	The National Institute on Aging.	No competing interests
Brodaty H & Low LF., 2004 [45]	Small sample size, lack of a CG.	The Alzheimer's Association of New South Wales.	No competing interests
Nobili A. et al., 2004 [48]	Losses of follow-up, short duration of the study.	Regione Lombardia, Assessorato alla Famiglia e Politiche Sociali.	No competing interests
Gitlin L et al., 2005 [29]	NS	The National Institute on Aging.	No competing interests
Graff M et al., 2006 [38]	Not double-blind study, sample might not be representative.	The Dutch Alzheimer Association, the Radboud University Nijmegen Medical Center and the Dutch Occupational Therapy Association.	No competing interests

Graff M et al., 2007 [39]	Not double-blind study, sample might not be representative.	The Dutch Alzheimer Association, the University Medical Center Nijmegen and the Dutch Occupational Therapy Association.	No competing interests
Onor ML et al., 2007 [49]	Small sample size, short duration of the study.	NS	No competing interests
Hilgeman M et al., 2007 [30]	Only compared longitudinal outcomes between White and African American caregivers at a single site across 12 months, the relative lack of attention to translation and the limited attention paid to the emotional outcomes of PwD.	NS	No competing interests
Chee KY et al.,2007 [31]	Interventionists provided the treatment implementation data, a potential source of response bias. Investigators restricted this study to caregivers who participated in four or more intervention sessions. Because most caregivers had the intended contact number, the relationship of dosage to adherence should be interpreted cautiously.	The National Institute on Aging Grant and by the Pennsylvania Department of Health Grant.	No competing interests
Gitlin L et al., 2008 [32]	The lack of an attention CG makes it impossible to exclude the possibility that demonstrated benefits are due to the intervention. Pilot studies may overestimate treatment benefit. Behavioural outcomes were reported by caregivers.	The National Institute of Mental Health.	No competing interests
Gitlin L et al., 2010 [33]	Inability to determine active treatment components, only 15% of study caregivers were male and a higher proportion of male caregivers than female caregivers dropped out.	The National Institute on Aging, the National Institute on Nursing Research and the Pennsylvania Department of Health, Tobacco Settlement.	No competing interests
Gitlin L. et al., 2010 [34]	Use of a no-treatment control group, the inability to disentangle effects of any one treatment component, IG dropouts reported less upset and behavioural symptoms than study completers.	The National Institute on Aging and the National Institute on Nursing Research Grant.	No competing interests
Voigt-Radloff S et al., 2011 [52]	Training time for the interventionists was short, interventionists had less treatment experience with the experimental intervention than the therapists of the Dutch original trial.	The German Federal Ministry of Health.	No competing interests
Sturkenboom I et al., 2013 [40]	Small sample size, a variation in amount and content of treatment sessions.	The Radboud University Nijmegen Medical Centre; Fonds Nuts Ohra and Prinses Beatrix Fonds/Parkinson Vereniging.	No competing interests
DiZazzo-Miller R et al., 2014 [35]	The use of different caregiver trainers at different sites.	The Faculty Research Award Program from the College of Pharmacy and Health Sciences, Wayne State University.	No competing interests

Sturkenboom I et al., 2014 [41]	The control group was not offered an intervention.	The Prinses Beatrix Spierfonds and the Parkinson Vereniging.	No competing interests
Gitlin L et al., 2018 [36]	Small sample size, use of a single veteran setting, medication change effects are unclear.	The Veterans Administration Health Services Research and Development Service	No competing interests
O'Connor C et al., 2019 [46]	Small simple size, biases may exist in the data.	ForeFront, from the National Health and Medical Research Council and the Australian Research Council. Alzheimer's Australia Dementia Research Foundation. The Alzheimer's Society.	No competing interests
De Oliveira A et al., 2019 [51]	Small simple size, the procedure for confirming the patient had dementia was based on medical reports, all patients randomized in the study were taking psychotropic medication.	The Alzira Denise Hertzog Silva Association, Instituto Nacional de Biomarcadores en Neuropsiquiatria, Sao Paulo Research Foundation, and National Council for Scientific and Technological Development.	No competing interests
Tang SH et al., 2018 [42]	Small simple size, heterogeneity across age, type and stage of dementia, individual caregiver differences in knowledge about active psychoeducation interventions.	The Taiwan Ministry of Health and Welfare, Executive Yuan, Taipei.	No competing interests
Lau WM et al., 2019 [43]	Small simple size, a lack of a CG, compliance of intervention was not measured.	The Medical Division of Tung Wah Group of Hospitals.	No competing interests
Novelli M et al., 2018 [50]	Small simple size, the caregivers who participated do not reflect the Brazilian demographic characteristics, low generalisability of results.	The São Paulo Research Foundation.	No competing interests
Lai FH et al., 2020 [44]	Social desirability bias, poorly standardized experimental design.	NS	No competing interests
Laver K et al., 2020 [47]	Inability to complete outcome assessments on all participants recruited, lack of follow-up.	The National Health and Medical Research Council in Australia.	No competing interests
DiZazzo-Miller R et al., 2020 [37]	Small simple size, low generalisability of results.	NS	No competing interests
Wenborn J et al., 2021 [53]	Low generalisability of results.	The National Institute for Health Research (NIHR) [Programme Grants for Applied Research (Grant Reference Number: RP-PG-0610-10108)], the NIHR Applied Research Collaboration (ARC) North Thames.	Work funded through NIHR research grant to M.O. (co-author).

CG, Control Group; IG, Intervention Group; NS, Not stated; PwD, People with dementia.
